# Mixed Lineage Leukemia 1 Promoted Neuron Apoptosis in Ischemic Penumbra *via* Regulating ASK-1/TNF-α Complex

**DOI:** 10.3389/fnana.2020.00036

**Published:** 2020-07-24

**Authors:** Zhang Feng, Liu Jie, Lv Guimin, Wang Xi

**Affiliations:** ^1^Department of Neurology, Shandong Provincial Western Hospital, Shandong Provincial ENT Hospital, Jinan, China; ^2^Department of Neurology, The Fourth Hospital of Jinan City, Jinan, China; ^3^Department of Neurology, Zibo Integrated Traditional Chinese and Western Medicine Hospital, Zibo, China; ^4^Department of Neurology, Chongqing Wulong Hospital of Traditional Chinese Medicine, Wulong, China

**Keywords:** mixed lineage leukemia 1, histone methylation, ischemic stroke, apoptosis, prognosis

## Abstract

Neuron apoptosis in ischemic penumbra was proved to be involved in ischemic stroke (IS) development and contributed to the poor prognosis of IS. Recent studies showed that aberrant trimethylation of histone H3 lysine 4 (H3K4me3) level was associated with cell apoptosis. This study aimed to explore the underlying mechanism of neuron apoptosis in ischemic penumbra *via* histone methyltransferase (HMT) mixed lineage leukemia 1 (MLL1) mediated epigenetic pathway. Mouse IS model was established by middle cerebral artery occlusion (MCAO). Mouse primary cortical mixed cells were cultured and treated with oxygen–glucose deprivation (OGD) to simulate IS process. The expressions of apoptosis signal regulating kinase-1 (ASK-1), pASK-1, cleaved caspase-3, ASK-1/serine–threonine kinase receptor-associated protein (STRAP)/14-3-3 complex, ASK-1/tumor necrosis factor-α (TNF-α) complex, and MLL1 in mouse brain tissue and mouse primary cortical mixed cells were analyzed. The function of MLL1 was investigated using small interfering RNA (siRNA) targeting MLL1 and vector overexpressing MLL1. *In vivo* inhibition of MLL1 was conducted to explore its value as a therapeutic target. The prognostic value of MLL1 was investigated in IS patients. Results showed that the expressions of ASK-1, pASK-1, cleaved caspase-3, ASK-1/TNF-α complex, and MLL1 increased significantly in ischemic penumbra compared to brain tissue from the control group (*P* < 0.05). MCAO and OGD significantly upregulated the H3K4me3 level in ASK-1 promoter region and promoted the recruitment of MLL1 to this region (*P* < 0.05). siMLL1 significantly reversed the proapoptosis effects of OGD in primary cortical mixed cells, while MLL1 overexpression induced apoptosis of cells (*P* < 0.05). *In vivo* inhibition of MLL1 significantly reduced the infarct volume and the neurological score of MCAO mice (*P* < 0.05). Serum MLL1 level had a positive association with that in ischemic core and penumbra in mouse model and was positively correlated with the infarct volume and neurological score (*P* < 0.05). Besides, serum MLL1 level was also significantly correlated with the severity of IS (*P* < 0.05), and high serum MLL1 level indicated poor prognosis of IS patients (*P* < 0.05). These results revealed that MLL1 contributed to neuron cell apoptosis in ischemic penumbra after IS onset by promoting the formation of ASK-1/TNF-α complex, and its serum level was associated with poor prognosis of IS.

## Introduction

Ischemic stroke (IS) is the most common type of stroke, accounting for ~80% of all strokes (Adeoye et al., 2019). The global mortality rate of IS is 10–40%, the recurrence rate of survivors is about 40%, and the disability rate is more than 50% (Guzik and Bushnell, 2017; Adeoye et al., 2019). Apoptosis of neuron cells in ischemic penumbra contributes a significant proportion to the development of acute brain ischemia, but the underlying mechanisms are still not fully understood (Radak et al., 2017).

Apoptosis signal regulating kinase 1 (ASK-1) is a component of tumor necrosis factor-α (TNF-α)-induced apoptosis complex I, and its activation (phosphorylation) is required for TNF-α-induced apoptosis in multiple cell types (Hatai et al., 2000; Han et al., 2015). To prevent TNF-α-induced apoptosis, serine–threonine kinase receptor-associated protein (STRAP) and 14-3-3 proteins interact with ASK-1 to disrupt associations between TNF receptor-associated factor 2 and ASK-1 upon TNF-α stimulation (Hatai et al., 2000; Han et al., 2015). In recent years, the aberrant histone methylation was suggested to be associated with neuron cell apoptosis (Zhoa et al., 2016; Yung et al., 2018). Histone methylation can promote or inhibit the transcription of methylation sites, and lysine (K) methylation of histone is a research hotspot in recent years (Alam et al., 2015; Kim et al., 2017). As an active histone modification, the trimethylation of H3K4 (H3K4me3) can be recognized by specific proteins and further recruit downstream coregulatory components, thus promoting the transcription and expression of the target genes (Bochyńska et al., 2018). Mixed lineage leukemia 1 (MLL1) is the main histone methyltransferase (HMT) responsible for the catalysis of H3K4me3 (Yang and Ernst, 2017), whose abnormal expression had been proven to be tightly associated with cell proliferation and apoptosis (Wang et al., 2014; Sengupta et al., 2019).

Based on the above background, we suggested a hypothesis that MLL1 was involved in neuron cell apoptosis in ischemic penumbra by regulating ASK-1-related apoptosis complexes. We explored the function and mechanism of MLL1 using mouse IS model and mouse primary cortical mixed cells. We also conducted a case–control study to explore the prognostic value of MLL1 in IS, thus providing a new approach to improve the prognosis of IS.

## Materials and Methods

### Ethics Approval

This study was approved by the Ethics Committee of the Shandong Provincial ENT Hospital affiliated to Shandong University (Shandong, China). Written informed consent was obtained from subjects following the Declaration of Helsinki. Animal experiments were all approved by the Shandong Provincial Key Laboratory of Otology (Shandong, China).

### *In vivo* Inhibition of MLL1

CD-1 mice (25–30 g, males) were purchased from the Charles River Labs (Beijing, China) and were housed in the Shandong Provincial Key Laboratory of Otology (Shandong, China). MM102 (HY-12220A, MCE), a specific WDR5/MLL interaction inhibitor, can significantly inhibit the catalytic activity of MLL1 (Hacer et al., 2013). Before surgery, MM102 was given by tail intravenous injection (0.10, 0.25, 0.50 mg/kg, three times a day) for 3 days. MM102 was dissolved in dimethyl sulfoxide (DMSO). Mouse injected with DMSO only was taken as the control.

### IS Model Establishment, Infarct Volume Measurement, Neurological Deficit Assessment, and Ischemic Penumbra Determination

Mice were randomly divided into the middle cerebral artery occlusion (MCAO) group and the control group. Mice from the MCAO group were anesthetized with chloral hydrate (300 mg/kg, i.p.) and fixed. The origin of the middle cerebral artery (MCA) was blocked by silicon-coated mono-filament nylon suture. The MCAO model was proved to be successful by the reduction in semicerebral blood flow shown by B-ultrasound detection. For mice from the control group, the same surgical procedure was performed except for the suture ligation of MCA. After the surgery, carprofen (5 mg/kg, three times a day) was given by subcutaneous injection for analgesia for 3 days. At 0, 1, 2, 4, 6, 12, 24, 36, and 48 h after modeling, 40 μl tail vein blood of each mouse was collected. Then, mice were euthanized. Brain tissues were quickly removed and sliced into sections of 2-mm thickness. The slices were stained with 0.5% triphenyl tetrazolium chloride (TTC) solution (Sh-haling Biotechnology, China) to show the infarct lesion (pale area). Infarct volume (%) = *V* (infarct area)/*V* (homolateral hemisphere) × 100%.

Neurological deficit score was assessed as previously described (Rogers et al., 1997) at 24 h after MCAO: (0) no neurological symptoms; (1) failure to extend right paw completely; (2) the strength of the right forelimb is noticeably reduced; (3) rotating and crawling towards the right side; and (4) unable to walk spontaneously. During the assessment, staffs were blind to the group information of each mouse.

**Figure 1:**
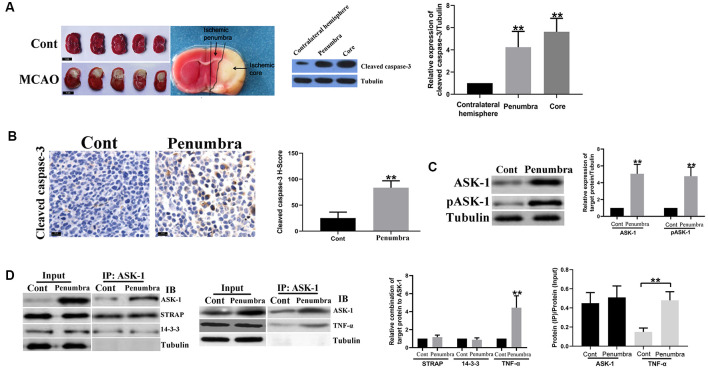
Apoptosis signal regulating kinase 1 (ASK-1)/tumor necrosis factor α (TNF-α) complex increased in ischemic penumbra. **(A)** Representative images of mice brain tissue in response to middle cerebral artery occlusion (MCAO) stained by triphenyl tetrazolium chloride (TTC) solution, and the spatial expression pattern of cleaved caspase-3 analyzed by Western blot. **(B)** Immunohistochemistry showed that the expression of cleaved caspase-3 increased significantly in ischemic penumbra from MCAO mice. **(C)** Western blot showed that total ASK-1 and pASK-1 increased significantly in the ischemic penumbra from MCAO mice. **(D)** Immunoprecipitation and western blot showed that the formation of ASK-1/TNF-α complex was enhanced in the ischemic penumbra from MCAO mice. Data are presented as mean ± SD, **P < 0.001. Mice were euthanized at 24 h after MACO, and there were 10 mice in each group.

According to the prior report (Ashwal et al., 1999), the middle coronal brain section was used for determining the ischemic penumbra. First, we made a longitudinal cut from the lateral of a sagittal suture through the right hemisphere, then made a transverse diagonal cut at approximately the “2 o’clock” position to separate the core from the penumbra. The infarction core was located in the lateral caudoputamen and the adjacent ventrolateral cortex of frontal–parietal, while the penumbra area was located in medial caudatoputamen and adjacent dorsal medial cortex of frontal–parietal (Figure 1A).

### Cell Culture and Treatment

The primary cortical mixed cells were obtained from mice gray matter. Briefly, the gray matter was collected and mechanically dissociated. Then, the tissues were digested by enzyme (0.55 mg collagenase per gram of initial brain tissue) at 37°C for 1 h. After centrifugation, cells were collected and cultured in high-glucose Dulbecco’s modified Eagle’s medium (DMEM; Gibco, USA) containing 10% fetal bovine serum (FBS; Gibco, USA), 100 U/ml penicillin, and 0.1 mg/ml streptomycin. Cells were placed in a 37°C incubator with 5% CO_2_. After 3 days incubation, oxygen–glucose deprivation (OGD) was performed as described previously (Zhou et al., 2019). Briefly, DMEM without glucose (Gibco, USA) was used to culture cells; then, cells were placed in a hypoxic environment (ThermoFisher Scientific, Waltham, MA, USA) with 1% O_2_, 5% CO_2_, and 94% N_2_ for different times. At 0, 1, 2, 4, 6, 12, 24, 36, and 48 h of OGD treatment, cells were collected and analyzed. Controls were cells maintained in normoxic conditions.

Brain microvascular endothelial cells (BMECs) were isolated from the gray matter of mice according to the previous report (Rosas-Hernandez et al., 2018). Briefly, the gray matter was collected and underwent mechanical and enzymatic digestion (0.55 mg collagenase per gram of initial brain tissue) for dissociation. After undergoing dextran separation and Percoll separation, cells were seeded on collagen-coated culture plates and cultured in DMEM containing 10% FBS for 3 days. The purity of BMECs was identified by immunostaining for factor VIII-related antigen/von Willebrand factor (vWF; Supplementary Figure S1A).

Cerebral astrocytes were also obtained from neonatal mice according to the previous report (Schildge et al., 2013). Briefly, meninges were removed and cortical pieces mechanically dissociated in culture medium (DMEM supplemented with 10% FBS). Dissociated cells were seeded into cell culture flasks. To obtain type 1 astrocytes, flasks with confluent cultures were shaken at 37°C overnight. Astrocytes were cultured in DMEM containing 10% FBS for 1 day. The purity of astrocytes was identified by immunostaining for glial fibrillary acidic protein (GFAP; Supplementary Figure S1A).

To construct *in vitro* model of the brain–blood barrier (BBB), we used a Transwell insert. Astrocytes (1.5 × 10^4^ cells/cm^2^) were seeded on the bottom side of the insert and were let to adhere firmly for overnight. Then, BMECs (1.5 × 10^5^ cells/cm^2^) were seeded in the upper side of the insert, while the primary cortical mixed cells were seeded in the plate under the insert (lower chamber; Supplementary Figure S1B). Two days after, MM102 (HY-12220A, MCE) was added to the upper chamber and incubated for 3 days. Then, the primary cortical mixed cells in the lower chamber were collected and analyzed. MM102 was dissolved in DMSO. Cells treated with DMSO only was taken as the control.

### Cell Transfection

Small interfering (siRNA; Genechem, China) targeting MLL1 and ASK-1 was purchased and used to silence MLL1 and ASK-1 expressions in primary cortical mixed cells, and the nontargeting siRNA (Genechem, China) was used as the negative control (NC). MLL1 and ASK-1 overexpression vectors (pG/CMV/MLL1/IRES/EGFP; pG/CMV/ASK-1/IRES/mCherry) were constructed and purchased (VectorBuilder, China), and the empty vectors were used as NC. Briefly, cells were cultured in culture without FBS for 24 h; then, the transfection was performed using Lipofectamine 3000 (Invitrogen, USA) according to the manufacturers’ instructions. After transfection, cells were placed in a hypoxic environment for another 24 h. The sequence of siRNA was provided in Supplementary Table S1.

### Western Blot Analysis

Cells were lysed with radioimmunoprecipitation assay (RIPA) lysate buffer (Beyotime, China). The same amount of protein was separated by 10% sodium dodecyl sulfate–polyacrylamide gel electrophoresis (SDS-PAGE) gel and then transferred to a polyvinyl difluoride (PVDF) membrane. After soaking in protein-free fast block buffer (Beyotime, China), the membrane was incubated overnight with one of the following antibodies at 4°C: anti-ASK-1 antibody (8662, Cell Signaling Technology), anti-pASK-1 antibody (3764, Cell Signaling Technology), anti-STRAP antibody (AP29336; One World Lab), anti-14-3-3 antibody (9636; Cell Signaling Technology), anticleaved caspase-3 antibody (ab2302, Abcam), anti-TNF-α antibody (ab1793, Abcam), anti-MLL1 antibody (ab32400, Abcam), anti-WDR5 antibody (ab178410, Abcam), and anti-tubulin antibody (ab6160, Abcam). Then, the secondary antibody was used to incubate the membrane at room temperature for 0.5 h. Finally, the membrane was observed by ECL Plus Kit (Beyotime, China) and quantified using the ImageJ program (National Institutes of Health, USA).

### Immunohistochemistry

Immunostaining was performed on paraffin-embedded sections. After blocking with 5% bovine serum albumin (BSA), tissues were incubated with the following antibodies overnight at 4°C: antibodies directed against MLL1 (ab32400, Abcam) and cleaved caspase-3 (9661S; Cell Signaling Technology) were used to detect the target proteins. All slides were visualized using DAB substrate (Beyotime, China) and quantified by *H* score. The *H* score was calculated using the following formula: *H* score = *Pi* (*i*), where *i* is the intensity of staining with a value of 1, 2, or 3 (weak, moderate, or strong, respectively) and *Pi* is the percentage of stained cells for each intensity, varying from 0 to 100%.

### Flow Cytometry Analysis

Cell apoptosis was assessed after OGD for 24 h using Annexin V fluorescein isothiocyanate (FITC)/apoptosis detection kit (Sigma, USA). Briefly, cells were resuspended and were incubated with 5 μl annexin V-fluorescein isothiocyanate (FITC) and 10 μl propidium iodide for 15 min in a dark room. Then, cells were analyzed using flow cytometer (BD Biosciences, USA). Data were analyzed using CellQuest (BD Biosciences, USA).

### Chromatin Immunoprecipitation Assay

Chromatin immunoprecipitation (ChIP) assay was performed using SimpleChIP^®^ Plus Sonication Chromatin IP Kit (56383, Cell Signaling Technology) according to the manufacturers’ instructions. Briefly, 50 μg chromatin was immunoprecipitated using 2 μg anti-MLL1 antibody (ab272023, Abcam), or 2 μg anti-H3K4me3 antibody (9751, Cell Signaling Technology). Immunoprecipitation with 2 μg normal rabbit immunoglobulin G (IgG) was used as the NC. Then, DNA was purified and was detected by quantitative PCR (qPCR). The primers used were provided in Supplementary Table S1.

### Immunoprecipitation

The lysates generated from mice brain tissues or primary cortical mixed cells were precleared with Protein G beads and followed by being incubated with 5 μg/ml anti-ASK-1 antibody (8662; Cell Signaling Technology) or anti-MLL1 antibody (ab32400, Abcam) overnight at 4°C. Then, Protein G beads were added and incubated for 2 h at 4°C. After that, beads were washed using lysis buffer for three times and were directly boiled in 1× Laemmli buffer. The coprecipitated proteins were further separated and analyzed by SDS-PAGE and Western blotting analysis.

### Real-Time Quantitative PCR

Total RNA was extracted from tissues or cells using Beyozol kit (Beyotime, China), and RNA concentration was determined by Nanodrop ND2000 (ThermoFisher Scientific, Waltham, MA, USA). Then, RNA was reversely transcribed into complementary DNA (cDNA) using PrimeScriptTM RT Master Mix (Takara, Japan). MLL1 messenger RNA (mRNA) was detected by BioRad CFX96 (BioRad Inc., USA) using SYBR Green qPCR Mix (CWBIO, China). MLL1 expression was normalized to glyceraldehyde-3-phosphate dehydrogenase (GAPDH) using 2^−ΔΔCt^ method. All the experiments were in triplicates. The sequence of primer was provided in Supplementary Table S1.

### Immunofluorescence

Cells were fixed with 4% polyoxymethylene for 10 min at room temperature and then were incubated with 5% BSA for 0.5 h at 37°C. After that, anti-MLL1 (14197; Cell Signaling Technology) and anticleaved caspase-3 antibody (05–765; Millipore Sigma), anti-GFAP antibody (ab7260, Abcam), or anti-vWF antibody (ab11713, Abcam) were added and incubated with cells overnight at 4°C, followed by incubating with secondary antibody (BA1101, BA1032, BOSTER) for another 0.5 h at 37°C. 2-(4-Amidinophenyl)-6-indolecarbamidine dihydrochloride was added to indicate the nucleus. Finally, cells were observed under an inverted microscope (Olympus, Japan).

### Human Study Subjects

Two hundred and twenty-three patients [148 men and 75 women; median age, 72.21; interquartile range (IQR), 63.32–82.54] diagnosed as IS between October 2015 and September 2019 at Shandong Provincial ENT Hospital affiliated to Shandong University (Shandong, China) were enrolled in this study. Patients were all diagnosed according to the results of magnetic resonance imaging and computed X-ray tomography. At admission, the National Institutes of Health Stroke Scale (NIHSS) score, the infarct volume, and the high-sensitivity C-reactive protein (Hs-CRP) were all assessed. One hundred and fifty age and gender-matched controls (100 men and 50 women; median age, 71.98; IQR, 65.63–77.88) from the physical examination center were also collected. Blood samples of IS patients were collected immediately at the admission [within 2–12 h (*n* = 74), 12–24 h (*n* = 106), 24–36 h (*n* = 31), and 36–48 h (*n* = 12) from symptom onset]. Blood samples of controls were collected at the time of physical examination.

### Follow-Up and Endpoints

After discharged from hospital, researchers followed patients up by telephone every half month for 24 months. The endpoint was the death within follow-up or patients quitting the study within 24 months. The modified Rankin Scale (mRS) of alive patients was obtained at 24 months after hospitalization at the outpatient department. An unfavorable functional outcome was defined as mRS scored more than 4 (Bonita and Beaglehole, 1988).

### Statistical Analysis

Statistical analyses were performed using SPSS version 20.0. For normally distributed data, results were shown as mean ± standard deviation (M ± SD), and for skewed data, results were presented as median (IQR). For differences between two groups (normally distributed variables), Student’s *t*-test was used, while one-way ANOVA was used for multiple groups. Categorical variables were analyzed by *χ*^2^ test. Correlations were analyzed using Pearson or Spearman correlation analysis. In this study, all experiments were repeated three times, and *P* < 0.05 was considered to be statistically significant.

## Results

### ASK-1/TNF-α Complex Increased in Ischemic Penumbra

Figure 1A showed mice brain sections in response to MCAO at 24 h and the region of ischemic core and penumbra. Compared to the contralateral hemisphere, cleaved caspase-3 elevated in ischemic core and penumbra, indicating that apoptosis existed in these two regions. Besides, compared to brain tissue from control mouse, cleaved caspase-3 was significantly upregulated in ischemic penumbra (Figure 1B), as well as the expressions of total ASK-1 and pASK-1 (Figure 1C). There is no difference in the formation of ASK-1/STRAP/14-3-3 complex (Figure 1D), while the formation of ASK-1/TNF-α complex increased markedly in ischemic penumbra (Figure 1D).

### ASK-1 Expression Was Regulated by MLL1

**Figure 2:**
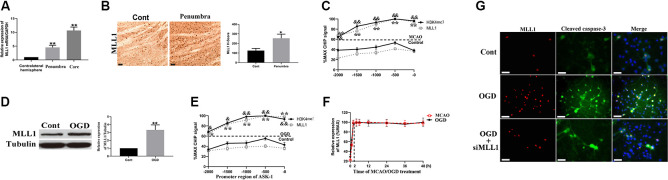
Apoptosis signal regulating kinase 1 (ASK-1) expression was regulated by mixed lineage leukemia 1 (MLL1). **(A)** Spatial expression pattern of MLL1 in MCAO mouse analyzed by quantitative PCR (qPCR). **(B)** Immunohistochemistry showed that the expression of MLL1 increased significantly in ischemic penumbra from MCAO mice. **(C)** Chromatin immunoprecipitation showed that the recruitment of MLL1 to ASK-1 promoter region was enhanced, and the H3K4me3 level in this region was increased. **(D)** Western blot showed that MLL1 expression increased significantly in primary cortical mixed cells from the oxygen–glucose deprivation (OGD) group. **(E)** Chromatin immunoprecipitation showed that OGD promoted the recruitment of MLL1 to the promoter region of ASK-1 and upregulated H3K4me3 level in this region. **(F)** qPCR indicated that MLL1 expression showed a rapid increase within the first 2 h after MACO/OGD, then kept a stable level within 2–48 h. **(G)** Immunofluorescence showed that OGD upregulated the expressions of MLL1 and cleaved caspase-3 in the same cell, and siMLL1 could reverse these effects. Data are presented as mean ± SD. Compared with MLL1 recruitment in control group, *P < 0.05, **P < 0.001; compared with H3K4me3 level in control group, ^&^*P* < 0.05, ^&&^*P* < 0.001. **(A–C)** Mice were euthanized 24 h after MACO. There were 10 mice in each group. **(D,E,G)** OGD was performed and lasted for 24 h. The scale bar indicates 5 μm.

We analyzed the spatial distribution of MLL1 in cerebral tissue of MCAO mouse. As the results showed, compared to the contralateral hemisphere, MLL1 expression was significantly upregulated in ischemic core and penumbra (Figure 2A). We next detected MLL1 expression in ischemic penumbra and mouse primary cortical mixed cells treated with OGD. Results showed that MLL1 expression was significantly upregulated in ischemic penumbra, as well as in cells treated with OGD (Figures 2B,D). CHIP assay showed that compared to control group, the recruitment of MLL1 to ASK-1 promoter region increased significantly in ischemic penumbra, as well as the H3K4 level in this region (Figure 2C). The same results were observed from mouse primary cortical mixed cells treated with OGD for 24 h (Figure 2E). The expression pattern of serum MLL1 in MCAO mice showed a rapid increase in the first 2 h, then kept a relatively stable level within 2–48 h, and the same expression pattern was observed in primary cortical mixed cells during OGD treatment (Figure 2F). Results of immunofluorescence showed that OGD could upregulate MLL1 and cleaved caspase-3 levels at the same cell, while siMLL1 could reverse these effects significantly (Figure 2G).

### MLL1 Was Essential for OGD-Induced Neuron Apoptosis

**Figure 3:**
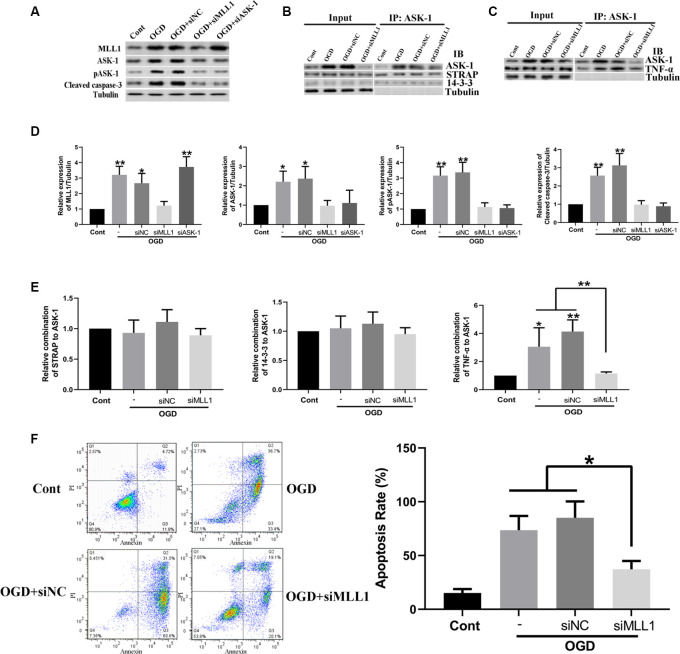
Loss of MLL1 alleviated neuron apoptosis by inhibiting apoptosis signal regulating kinase 1 (ASK-1)/tumor necrosis factor α (TNF-α) complex formation. **(A)** Western blot showed that OGD significantly upregulated the expressions of MLL1, total ASK-1, pASK-1, and cleaved caspase-3 in primary cortical mixed cells, while these effects could be blocked by siMLL1 and siASK-1. **(B–E)** Immunoprecipitation showed that no significant difference was found in the formation of ASK-1/STRAP/14-3-3 complex among different groups, while OGD significantly enhanced the formation of ASK-1/TNF-α complex in primary cortical mixed cells, and this effect could be blocked by siMLL1. **(F)** Flow cytometry analysis showed that OGD promoted the apoptosis of primary cortical mixed cells, while this effect could be blocked by siMLL1. Data are presented as mean ± SD, *P < 0.05, **P < 0.001. OGD was performed and lasted for 24 h.

**Figure 4:**
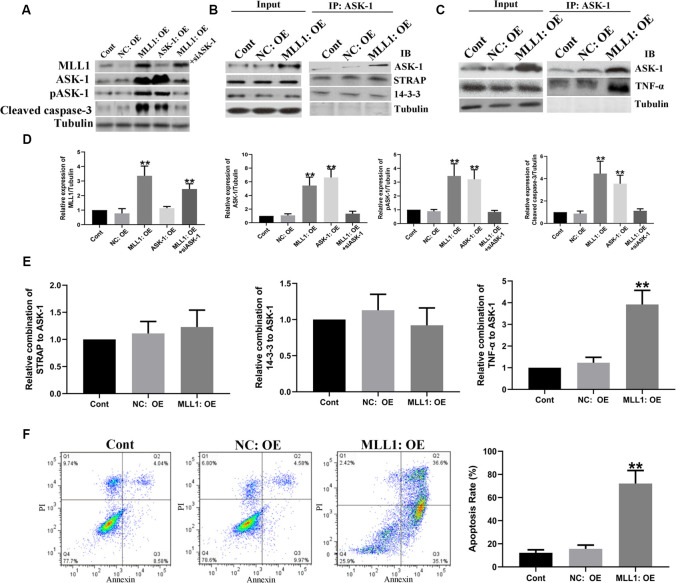
Gain of MLL1 promoted neuron apoptosis by enhancing apoptosis signal regulating kinase 1 (ASK-1)/tumor necrosis factor α (TNF-α) complex formation. **(A)** Western blot showed that overexpression of MLL1 and ASK-1 significantly upregulated the expressions of total ASK-1, pASK-1, and cleaved caspase-3 in primary cortical mixed cells, while siASK-1 blocked the effects induced by MLL1 overexpression. **(B–E)** Immunoprecipitation showed that no significant difference was found in the formation of ASK-1/STRAP/14-3-3 complex among different groups, while MLL1 overexpression significantly enhanced the formation of ASK-1/TNF-α complex in primary cortical mixed cells. **(F)** Flow cytometry analysis showed that MLL1 overexpression promoted the apoptosis of primary cortical mixed cells. Data are presented as mean ± SD, **P < 0.001. OGD was performed and lasted for 24 h.

Mouse primary cortical mixed cells were used to investigate the function of MLL1. As the results showed, OGD significantly upregulated the expression of MLL1, total ASK-1, pASK-1, and cleaved caspase-3, while siMLL1 and siASK-1 significantly reversed these effects. OGD enhanced the formation of ASK-1/TNF-α complex to promote cell apoptosis, and siMLL1 reversed the effects of OGD (Figure 3). Besides, overexpression of MLL1 and ASK-1 significantly increased total ASK-1, pASK-1, and cleaved caspase-3 expressions in primary cortical mixed cells, while siASK-1 blocked these effects induced by MLL1 overexpression. Overexpression of MLL1 further enhanced the formation of ASK-1/TNF-α complex and promoted apoptosis of primary cortical mixed cells (Figure 4).

### Inhibition of MLL1 Alleviated Neurological Damage Caused by IS

**Figure 5:**
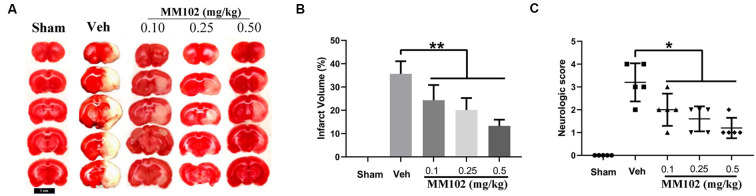
Inhibition of MLL1 alleviated neurological damage caused by ischemic stroke (IS). **(A)** Representative images of mice brain tissue in response to MCAO and MM102 stained by TTC solution. **(B,C)** MM102 treatment reduced the infarct volume and the neurological score of MCAO mice. Data are presented as mean ± SD, *P < 0.05, **P < 0.001. Mice were euthanized 24 h after MACO. There were 10 mice in each group.

We first analyzed whether MM102 could cross the BBB using an *in vitro* BBB model. The results showed that MM102 could penetrate the barrier consisting of BMECs and astrocytes to inhibit the interaction between MLL1 and WDR5 (Supplementary Figure S1C). Next, we designed three different concentrations of MM102 to investigate the effects of MLL1 inhibition *in vivo*. As the results showed, compared to the Veh group, using MM102 could significantly decrease infarct volume and the neurological score of MCAO mice (Figure 5).

### Serum MLL1 Level Was Significantly Correlated With the Severity of IS

**Figure 6:**
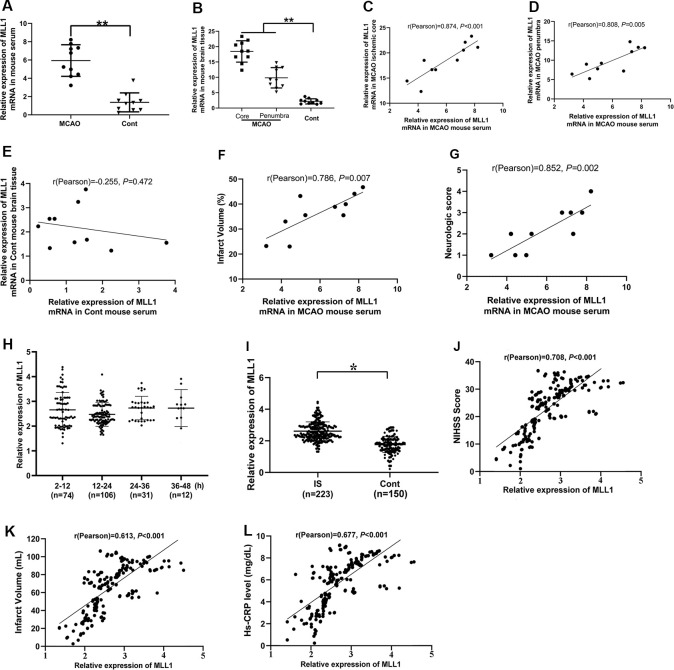
Serum MLL1 level was significantly correlated with the severity of IS. **(A,B)** MLL1 level in MCAO mouse serum, ischemic core, and ischemic penumbra were all significantly higher than that in the control mouse. **(C–G)** MLL1 level in MCAO mouse serum was positively correlated with that in the ischemic core and penumbra, and it was also positively correlated with infarct volume and neurological score. **(H)** No difference was found in serum MLL1 level among patients admitted to hospital at different times after IS onset. **(I)** Serum MLL1 level of IS patients was significantly higher than that of control. **(J–L)** Serum MLL1 level was positively correlated with NIHSS score, infarct volume, and serum high-sensitivity C-reactive protein (Hs-CRP) level of IS patients. Data are obtained using qPCR and presented as mean ± SD, *P < 0.05, **P < 0.001. Mice were euthanized at 24 h after MACO. There were 10 mice in each group.

MLL1 expressions in mouse serum and brain tissue were detected by qPCR. As the results showed, MLL1 levels in both serum and brain tissues from the MCAO group were significantly higher than that in the control group (Figures 6A,B). Correlation analysis was performed to explore the association between MLL1 levels in serum and brain tissue. Results showed that serum MLL1 level in MCAO mouse was significantly correlated with that in ischemic core and penumbra (*r* = 0.874, *P* < 0.001, Figure 6C; *r* = 0.808, *P* = 0.005, Figure 6D), but not in normal cerebral tissue from control mice (*r* = −0.255, *P* = 0.472, Figure 6E). Besides, MCAO mouse serum MLL1 level was also positively correlated with the infarct volume and neurological score (*r* = 0.786, *P* = 0.007, Figure 6F; *r* = 0.852, *P* = 0.002, Figure 6G).

We next analyzed serum MLL1 level in IS patients admitted to the hospital at different times, and no significant difference was found (Figure 6H; *P* = 0.428). However, we did find that the serum MLL1 level in IS patients was significantly higher than in controls (Figure 6I, *P* = 0.013). Correlation analysis showed that serum MLL1 level had no association with age, gender, and the disease subtypes (Supplementary Table S2) but was significantly correlated with disease severity. Seum MLL1 level was positively correlated with NIHSS score (*r* = 0.708, *P* < 0.001, Figure 6J), infarct volume (*r* = 0.613, *P* < 0.001, Figure 6K), and Hs-CRP level (*r* = 0.677, *P* < 0.001, Figure 6L).

### High MLL1 Expression Indicated Poor Prognosis of IS Patients

**Figure 7:**
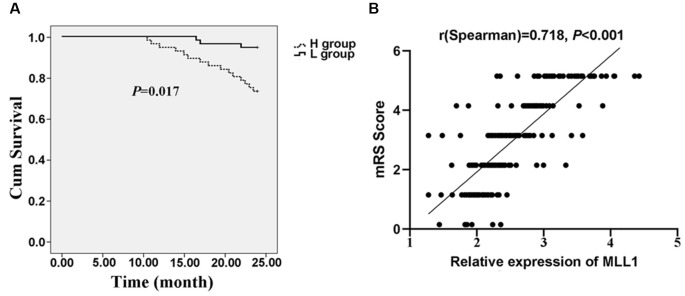
The prognostic value of MLL1. **(A)** High serum MLL1 level indicated higher mortality of IS patients within 24 months. **(B)** Serum MLL1 level was positively correlated with modified Rankin Scale (mRS) score at 24 months after IS onset.

Two hundred twenty-three patients discharged from the hospital were enrolled in the follow-up. Among them, 37 patients died within 24 months and three quitted the study. We divided patients into the high MLL1 group (H group) and the low MLL1 level group (L group) according to the median level of MLL1. Compared to patients with high MLL1 level, patients in L group had a significantly higher survival rate (*P* = 0.017, Figure 7A). For live patients, mRS was obtained at 24 months after hospitalization at the outpatient department. Results showed that high serum MLL1 level indicated an unfavorable functional outcome of IS patients within 24 months (*r* = 0.718, *P* < 0.001, Figure 7B).

## Discussion

In the present study, we found that MLL1 was upregulated in the ischemic penumbra and OGD-treated primary cortical mixed cells, and its expression level in ischemic penumbra was positively correlated with its serum level and also positively correlated with the severity of IS. *In vitro* study revealed that OGD promoted the recruitment of MLL1 to the promoter region of ASK-1 and enhanced the activation of ASK-1/TNF-α-mediated apoptosis. In addition, our study also suggested that high MLL1 levels in the serum indicated higher mortality and a poorer functional outcome of IS patients within 24 months.

Concerning the potential mechanism of IS development, it is still not fully clarified. IS can be caused by brain ischemia. The blockage of a brain artery accounts for ~80% of the IS cases (Adeoye et al., 2019). In the process of IS development, the cerebral tissue has two important and independent areas: the core of the infarct and the ischemic penumbra. The core of the infarct is defined as the severe hypoperfusion area where neurons directly affected by the lack of glucose and oxygen, and eventually die by necrosis, while the ischemic penumbra is the less hypoperfused area surrounding the core, where neuronal cells are still metabolically active within a certain period and—depending on circumstances—will either die or survive (Berta et al., 2018). Cell apoptosis in the ischemic penumbra may occur in hours or days, and this process is multifactorial and complex (Radak et al., 2017). Presently, local recanalization and systemic thrombolysis are the only therapeutic options to treat IS, with a short therapeutic window for 4.5 h after stroke onset, and only 20% of patients benefit from this treatment (Chamorro et al., 2016). Finding mechanisms of neuron apoptosis in ischemic penumbra is a key way to improve the prognosis of IS patients.

Recently, aberrant epigenetic histone modification was found to be involved in neuron apoptosis. As an active histone modification, the H3K4me3 level had been proven to be upregulated in the promoter region of some specific genes during the neuronal apoptotic process (Yung et al., 2018). MLL1 is the main HMT that catalyzes the methylation of H3K4 site (Yang and Ernst, 2017). MLL1 had drawn people’s attention because of its function in the development of mixed lineage leukemia (Cao et al., 2014). However, concerning MLL1 and IS, the related document is extremely limited.

In the present study, for the first time, we explored the function of MLL1 in neuron apoptosis in ischemic penumbra. We found that MLL1 increased significantly in ischemic penumbra after IS onset, and the upregulated MLL1 was recruited to the promoter region of ASK-1 to catalyze H3K4me3 in this region and further promoted the expression of ASK-1 and enhanced the activation of ASK-1/TNF-α-mediated apoptosis. ASK-1 is a cytosolic protein, and TNF-α is an extracellular protein; these two proteins interacted indirectly based on their interactions with the tumor necrosis factor receptor type 1 (TNFR1; Han et al., 2015). TNFR1 is a transmembrane molecule. After the initiation of TNFR1, the TNFR1-associated death domain (TRADD) protein is shuttled from TNFR1 to the cytoplasm and then interacts with the Fas-associated *via* death domain (FADD) protein and caspase to generate apoptosis complex to amplify TNF-α-induced apoptosis (Micheau and Jürg, 2003). MM102, a small-molecule inhibitor, was further used to investigate the function of MLL1. We built an *in vitro* model to estimate whether MM102 could cross the BBB. *In vitro* BBB models have been in use for decades and have been of great benefit in the process of investigating and understanding the cellular and molecular mechanisms underlying BBB establishment. However, there are still some differences between the *in vitro* model of BBB and the *in vivo* one (Czupalla et al., 2014). First, BMECs are known to dedifferentiate *in vitro*, and the characteristics of BMECs will gradually disappear with the extension of culture time; then, the function of these cells will be affected. Second, the *in vivo* BBB is composed of BMECs, astrocytes, and pericytes; however, the *in vitro* model we used in this study only consists of BMECs and astrocytes and that may cripple the barrier function of BBB. Third, the *in vitro* BBB models always rely on Transwell chambers for functional measurements, while BMECs behave differently according to their growth surface, so the different cultural materials may lead to different results. Due to these defects, the results should be explained cautiously. In our study, results of the *in vitro* study showed that MM102 could cross the BBB, and *in vivo* inhibition of MLL1 alleviated neurological damage caused by IS; these both suggest that MM102 is an effective molecule for IS treatment.

Additionally, we observed that serum MLL1 level was positively correlated with that in ischemic penumbra from MCAO mouse, indicating the feasibility to detect MLL1 by minimally invasive means. Since serum MLL1 level was positively correlated with that in ischemic core and penumbra in MCAO mice, we speculated that MLL1 was released from the damaged neurocytes into the blood. MLL1 mRNA in the serum might also be from the damaged vessels, blood cells, and/or cells in other organs affected by the systemic inflammatory response (Anrather and Costantino, 2016). What is more, we found that MLL1 expression showed a rapid increase within the primary 2 h after IS onset and remained a stable level within 2–48 h. This will promise us plenty of time to diagnose patients, and it also suggests that detecting MLL1 in IS patients is feasible and credible. To verify the prognostic value of MLL1, we followed up 220 patients, and the results revealed that high serum MLL1 level indicated higher mortality and a poorer functional outcome, and these results were consistent with the results that serum MLL1 level was positively correlated with NIHSS scores, infarct volume, and Hs-CRP level of IS patients.

In conclusion, the current study explored the function of MLL1 in neuron apoptosis and found that MLL1 served as a proapoptosis factor in the ischemic penumbra. Besides, serum MLL1 level is an effective indicator to reflect disease severity and to indicate the prognosis of IS. These results may provide us the theoretical basis for a new therapeutic target for IS.

## Data Availability Statement

The raw data supporting the conclusions of this article will be made available by the authors, without undue reservation, to any qualified researcher.

## Ethics Statement

The studies involving human participants were reviewed and approved by Ethics Committee of Shandong Provincial ENT Hospital affiliated to Shandong University. The patients/participants provided their written informed consent to participate in this study. The animal study was reviewed and approved by Shandong Provincial Key Laboratory of Otology.

## Author Contributions

WX developed the hypothesis and designed the research. ZF and LJ executed the experiments. ZF and LG participated in data analysis and manuscript writing.

## Conflict of Interest

The authors declare that the research was conducted in the absence of any commercial or financial relationships that could be construed as a potential conflict of interest.
