# Supplementary material for: Mixed Lineage Leukemia 1 Promoted Neuron Apoptosis in Ischemic Penumbra via Regulating ASK-1/TNF-α Complex
Source: Front Neuroanat. 2020 Jul 24;14:36. doi: 10.3389/fnana.2020.00036 (PMC7394220; doi:10.3389/fnana.2020.00036)
Supplement: Supplementary file 1 [file Image_1.pdf]

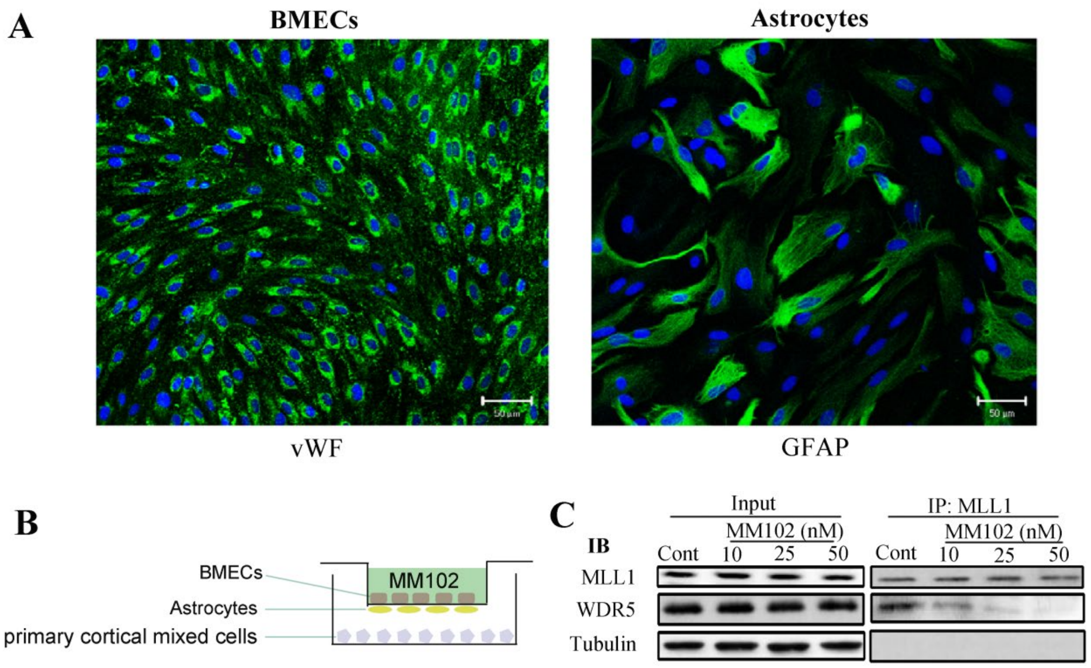

Supplementary Figure 1. A. The identification of the purity of primary BMECs and astrocytes. B. Schematic of the *in vitro* brain-blood barrier model. C. Representative images of immunoprecipitation and western blot showed that MM102 could cross the barrier consisting of BMECs and astrocytes to inhibit the interaction between MLL1 and WDR5.
