# Supplementary material for: Mixed Lineage Leukemia 1 Promoted Neuron Apoptosis in Ischemic Penumbra via Regulating ASK-1/TNF-α Complex
Source: Front Neuroanat. 2020 Jul 24;14:36. doi: 10.3389/fnana.2020.00036 (PMC7394220; doi:10.3389/fnana.2020.00036)
Supplement: Supplementary file 2 [file Table_1.DOCX]

Supplementary Table 1. Sequence of siRNA and primer

|  | **Name** | **Forward/** **Sense (from 5’ to 3’)** | **Reverse/Antisense (from 5’ to 3’)** |
| --- | --- | --- | --- |
| siRNA | siMLL1 | CTGCCGATGCCTACGUGCGCCTACG | CGTCGCGTCGAUGCAGCAGCTCA |
|  | siNC | UUCUCCGAACGUGUCACGUTT | ACGUGACACGUUCGGAGAATT |
|  | siASK-1 | CATCCCATGCTTTCCUUCGCTTCTG | CCGTTGCCATGCATTGCCGTA |
| Gene | MLL1 | TGGTGCTGACTCCACATACG | AGGTGGTGACCCCAACATTC |
|  | GAPDH | CAGGTTGTCTCCTGCGACTT | TATGGGGGTCTGGGATGGAA |
| Promoter of ASK-1 | -1983~-1855 | TGCAGCAGGAACGTGGTAAT | CATCGCTGTTTCCATCGCAG |
|  | -1642~-1486 | TGCGCCAGCTTGGTTATACA | AACGTCTCCAGCACATTCGT |
|  | -1103~-956 | TTTACAGAGCCCACATGCGT | ACTGTGTACCACTTCGCCAG |
|  | -622~-475 | TCACTACGGCGTTAGGGAGA | GGCCAGGTAAACACTTCCGA |
|  | -97~36 | AACAGCGATGGATGTGGTGT | TGTATAACCAAGCTGGCGCA |

Supplementary Table 2. The correlation between serum MLL1 level and the clinical characteristics of IS patients

| **Clinical characteristics** | **n** | **MLL1** | ***t/F*** | ***P*** |
| --- | --- | --- | --- | --- |
| Gender |  |  | 1.244 | 0.658 |
| male | 148 | 2.75±0.53 |  |  |
| female | 75 | 2.57±0.44 |  |  |
| Age |  |  | 0.833 | 0.596 |
| <60 | 64 | 2.81±0.61 |  |  |
| ≥60 | 159 | 2.64±0.43 |  |  |
| Disease subtype |  |  | 1.023 | 0.701 |
| Large-artery atherosclerosis | 122 | 2.63±0.59 |  |  |
| Cardioembolism | 59 | 2.67±0.48 |  |  |
| Small vessel occlusion | 42 | 2.88±0.51 |  |  |
